# Models in biology: ‘accurate descriptions of our pathetic thinking’

**DOI:** 10.1186/1741-7007-12-29

**Published:** 2014-04-30

**Authors:** Jeremy Gunawardena

**Affiliations:** 1 Department of Systems Biology, Harvard Medical School, 200 Longwood Avenue, Boston, USA

**Keywords:** Mathematical model, Predictive model, Fundamental physical laws, Phenomenology, Membrane-bounded compartment, T-cell receptor, Somitogenesis clock

## Abstract

In this essay I will sketch some ideas for how to think about models in biology. I will begin by trying to dispel the myth that quantitative modeling is somehow foreign to biology. I will then point out the distinction between forward and reverse modeling and focus thereafter on the former. Instead of going into mathematical technicalities about different varieties of models, I will focus on their logical structure, in terms of assumptions and conclusions. A model is a logical machine for deducing the latter from the former. If the model is correct, then, if you believe its assumptions, you must, as a matter of logic, also believe its conclusions. This leads to consideration of the assumptions underlying models. If these are based on fundamental physical laws, then it may be reasonable to treat the model as ‘predictive’, in the sense that it is not subject to falsification and we can rely on its conclusions. However, at the molecular level, models are more often derived from phenomenology and guesswork. In this case, the model is a test of its assumptions and must be falsifiable. I will discuss three models from this perspective, each of which yields biological insights, and this will lead to some guidelines for prospective model builders.

## The revenge of Erwin Chargaff

When I first came to biology from mathematics, I got used to being told that there was no place for mathematics in biology. Being a biological novice, I took these strictures at face value. In retrospect, they proved helpful because the skepticism encouraged me to let go of my mathematical past and to immerse myself in experiments. It was only later, through having to stand up in front of a class of eager students and say something profound (I co-teach Harvard’s introductory graduate course in Systems Biology), that I realized how grievously I had been misled. Biology has some of the finest examples of how quantitative modeling and measurement have been used to unravel the world around us [1,2]. The idea that such methods would not be used would have seemed bizarre to the biochemist Otto Warburg, the geneticist Thomas Hunt Morgan, the evolutionary biologist R. A. Fisher, the structural biologist Max Perutz, the stem-cell biologists Ernest McCulloch and James Till, the developmental biologist Conrad Waddington, the physiologist Arthur Guyton, the neuroscientists Alan Hodgkin and Andrew Huxley, the immunologist Niels Jerne, the pharmacologist James Black, the epidemiologist Ronald Ross, the ecologist Robert MacArthur and to others more or less well known.

Why is it that biologists have such an odd perception of their own discipline? I attribute this to two factors. The first is an important theme in systems biology [3,4]: the mean may not be representative of the distribution. Otto Warburg is a good example. In the eyes of his contemporaries, Warburg was an accomplished theorist: ‘to develop the mathematical analysis of the measurements required very exceptional experimental and theoretical skill’ [5]. Once Warburg had opened the door, however, it became easy for those who followed him to avoid acquiring the same skills. Of Warburg’s three assistants who won Nobel Prizes, one would not describe Hans Krebs or Hugo Theorell as ‘theoretically skilled’, although Otto Meyerhoff was certainly quantitative. On average, theoretical skills recede into the long tail of the distribution, out of sight of the conventional histories and textbooks. It is high time for a revisionist account of the history of biology to restore quantitative reasoning to its rightful place.

The second factor is the enormous success of molecular biology. This is ironic, for many of the instigators of that revolution were physicists: Erwin Schrödinger, Max Delbrück, Francis Crick, Leo Szilard, Seymour Benzer and Wally Gilbert. There was, in fact, a brief window, during the life of physicist George Gamow’s RNA Tie Club, when it was claimed, with poor judgment, that physics and information theory could work out the genetic code [6,7]. Erwin Chargaff, who first uncovered the complementarity of the A-T and G-C nucleotide pairs (Chargaff’s rules), was nominally a member of the club—his code name was lysine—but I doubt that he was taken in by such theoretical pretensions. He famously described the molecular biology of the time as ‘the practice of biochemistry without a license’ [8]. When Marshall Nirenberg and Heinrich Matthaei came out of nowhere to make the first crack in the genetic code [9], thereby showing that licensing was mandatory—one can just sense the smile on Chargaff’s face—the theorists of the day must have felt that the barbarians were at the gates of Rome. Molecular biology never recovered from this historic defeat of theory and there have been so many interesting genes to characterize since, it has never really needed to.

It is the culmination of molecular biology in the genome projects that has finally brought diminishing returns to the one gene, ten PhDs way of life. We now think we know most of the genes and the interesting question is no longer characterizing this or that gene but, rather, understanding how the various molecular components collectively give rise to phenotype and physiology. We call this systems biology. It is a very different enterprise. It has brought into biology an intrusion of aliens and concepts from physics, mathematics, engineering and computer science and a renewed interest in the role of quantitative reasoning and modeling, to which we now turn.

## Forward and reverse modeling

We can distinguish two kinds of modeling strategy in the current literature. We can call them forward and reverse modeling. Reverse modeling starts from experimental data and seeks potential causalities suggested by the correlations in the data, captured in the structure of a mathematical model. Forward modeling starts from known, or suspected, causalities, expressed in the form of a model, from which predictions are made about what to expect.

Reverse modeling has been widely used to analyze the post-genome, -omic data glut and is sometimes mistakenly equated with systems biology [10]. It has occasionally suggested new conceptual ideas but has more often been used to suggest new molecular components or interactions, which have then been confirmed by conventional molecular biological approaches. The models themselves have been of less significance for understanding system behavior than as a mathematical context in which statistical inference becomes feasible. In contrast, most of our understanding of system behavior, as in concepts such as homeostasis, feedback, canalization and noise, have emerged from forward modeling.

I will focus below on the kinds of models used in forward modeling. This is not to imply that reverse modeling is unimportant or uninteresting. There are many situations, especially when dealing with physiological or clinical data, where the underlying causalities are unknown or hideously complicated and a reverse-modeling strategy makes good sense. But the issues in distilling causality from correlation deserve their own treatment, which lies outside the scope of the present essay [11].

## The logical structure of models

Mathematical models come in a variety of flavors, depending on whether the state of a system is measured in discrete units (‘off’ and ‘on’), in continuous concentrations or as probability distributions and whether time and space are themselves treated discretely or continuously. The resulting menagerie of ordinary differential equations, partial differential equations, delay differential equations, stochastic processes, finite-state automata, cellular automata, Petri nets, hybrid models,... each have their specific technical foibles and a vast associated technical literature. It is easy to get drowned by these technicalities, while losing sight of the bigger picture of what the model is telling us. Underneath all that technical variety, each model has the same logical structure.

Any mathematical model, no matter how complicated, consists of a set of assumptions, from which are deduced a set of conclusions. The technical machinery specific to each flavor of model is concerned with deducing the latter from the former. This deduction comes with a guarantee, which, unlike other guarantees, can never be invalidated. Provided the model is correct, if you accept its assumptions, you must as a matter of logic also accept its conclusions. If ‘Socrates is a man’ and ‘All men are mortal’ then you cannot deny that ‘Socrates is mortal’. The deductive process that leads from assumptions to conclusions involves much the same Aristotelian syllogisms disguised in the particular technical language appropriate to the particular flavor of model being used or, more often, yet further disguised in computer-speak. This guarantee of logical rigor is a mathematical model’s unique benefit.

Note, however, the fine print: ‘provided the model is correct’. If the deductive reasoning is faulty, one can draw any conclusion from any assumption. There is no guarantee that a model is correct (only a guarantee that if it is correct then the conclusions logically follow from the assumptions). We have to hope that the model’s makers have done it right and that the editors and the reviewers have done their jobs. The best way to check this is to redo the calculations by a different method. This is rarely easy but it is what mathematicians do within mathematics itself. Reproducibility improves credibility. We may not have a guarantee that a model is correct but we can become more (or less) confident that it is. The practice of mathematics is not so very different from the experimental world after all.

The correctness of a model is an important issue that is poorly addressed by the current review process. However, it can be addressed as just described. From now on, I will assume the correctness of any model being discussed and will take its guarantee of logical validity at face value.

The guarantee tells us that the conclusions are already wrapped up in the assumptions, of which they are a logical consequence. This is not to say that the conclusions are obvious. This may be far from the case and the deductive process can be extremely challenging. However, that is a matter of mathematical technique. It should not distract from what is important for the biology, which is the set of assumptions, or the price being paid for the conclusions being drawn. Instead of asking whether we believe a model’s conclusions, we should be asking whether we believe the model’s assumptions. What basis do we have for doing so?

## On making assumptions

Biology rests on physics. At the length scales and timescales relevant to biology, physicists have worked out the fundamental laws governing the behavior of matter. If our assumptions can be grounded in physics, then it seems that our models should be predictive, in the sense that they are not subject to falsification—that issue has already been taken care of with the fundamental laws—so that we can be confident of the conclusions drawn. Physicists would make an even stronger claim on the basis that, at the fundamental level, there is nothing other than physics. As Richard Feynman put it, ‘all things are made of atoms and... everything that living things do can be understood in terms of the jigglings and wigglings of atoms’ [12, Chapter 3-3]. This suggests that provided we have included all the relevant assumptions in our models then whatever is to be known should emerge from our calculations. Models based on fundamental physical laws appear in this way to be objective descriptions of reality, which we can interrogate to understand reality. This vision of the world and our place in it has been powerful and compelling.

Can we ground biological models on fundamental physical laws? The Schrödinger equation even for a single protein is too hideously complicated to solve directly. There is, however, one context in which it can be approximated. Not surprisingly, this is at the atomic scale of which Feynman spoke, where molecular dynamics models can capture the jigglings and wigglings of the atoms of a protein in solution or in a lipid membrane in terms of physical forces [13]. With improved computing resources, including purpose-built supercomputers, such molecular dynamics models have provided novel insights into the functioning of proteins and multi-protein complexes [14,15]. The award of the 2013 Nobel Prize in Chemistry to Martin Karplus, Michael Levitt and Arieh Warshel recognizes the broad impact of these advances.

As we move up the biological scale, from atoms to molecules, we enter a different realm, of chemistry, or biochemistry, rather than physics. But chemistry is grounded in physics, is it not? Well, so they say but let us see what actually happens when we encounter a chemical reaction

A+B→C

and want to study it quantitatively. To determine the rate of such a reaction, the universal practice in biology is to appeal to the law of mass action, which says that the rate is proportional to the product of the concentrations of the reactants, from which we deduce that

$$\frac{d[\text{C}]}{\text{dt}}=k[\text{A}]\;[\text{B}],$$

where [-] denotes concentration and *k* is the constant of proportionality. Notice the immense convenience that mass action offers, for we can jump from reaction to mathematics without stopping to think about the chemistry. There is only one problem. This law of mass action is not chemistry. A chemist might point out, for instance, that the reaction of hydrogen and bromine in the gas phase to form hydrobromic acid,

$${\text{H}}_{2}+{\text{Br}}_{2}\to 2\text{HBr},$$

has a rate of reaction given by

$$\frac{d[\;\text{HBr}]}{\text{dt}}=\frac{k_{1}[\;{\text{H}}_{2}]{\left[\;{\text{Br}}_{2}\right]}^{3/2}}{\left[\;{\text{Br}}_{2}]+k_{2}[\;\text{HBr}\right]},$$

which is rather far from what mass action claims, and that, in general, you cannot deduce the rate of a reaction from its stoichiometry [16]. (For more about the tangled tale of mass action, see [17], from which this example is thieved.) Mass action is not physics or even chemistry, it is phenomenology: a mathematical formulation, which may account for observed behavior but which is not based on fundamental laws.

Actually, mass action is rather good phenomenology. It has worked well to account for how enzymes behave, starting with Michaelis and Menten and carrying on right through to the modern era [18]. It is certainly more principled than what is typically done when trying to convert biological understanding into mathematical assumptions. If *A* is known to activate *B*—perhaps *A* is a transcription factor and *B* a protein that is induced by *A*—then it is not unusual to find activation summarized in some Hill function of the form

(1) $$\frac{d[\;\text{B}]}{\text{dt}}=\frac{M{\left[\;\text{A}\right]}^{h}}{K^{h}+{\left[\;\text{A}\right]}^{h}},$$

for which, as Hill himself well understood and has been repeatedly pointed out [19], there is almost no realistic biochemical justification. It is, at best, a guess.

The point here is not that we should not guess; we often have no choice but to do so. The point is to acknowledge the consequences of phenomenology and guessing for the kinds of models we make. They are no longer objective descriptions of reality. They can no longer be considered predictive, in the sense of physics or even of molecular dynamics. What then are they?

One person who understood the answer was the pharmacologist James Black [20]. Pharmacology has been a quantitative discipline almost since its inception and mathematical models have formed the basis for much of our understanding of how drugs interact with receptors [21]. (Indeed, models were the basis for understanding that there might be such entities as receptors in the first place [2]). Black used mathematical models on the road that led to the first beta-adrenergic receptor antagonists, or beta blockers, and in his lecture for the 1988 Nobel Prize in Physiology or Medicine he crystallized his understanding of them in a way that nobody has ever bettered: ‘Models in analytical pharmacology are not meant to be descriptions, pathetic descriptions, of nature; they are designed to be accurate descriptions of our pathetic thinking about nature’ [22]. Just substitute ‘systems biology’ for ‘analytical pharmacology’ and you have it. Black went on to say about models that: ‘They are meant to expose assumptions, define expectations and help us to devise new tests’.

An important difference arises between models like this, which are based on phenomenology and guesswork, and models based on fundamental physics. If the model is not going to be predictive and if we are not certain of its assumptions, then there is no justification for the model other than as a test of its (pathetic) assumptions. The model must be falsifiable. To achieve this, it is tempting to focus on the model, piling the assumptions up higher and deeper in the hope that they might eventually yield an unexpected conclusion. More often than not, the conclusions reached in this way are banal and unsurprising. It is better to focus on the biology by asking a specific question, so that at least one knows whether or not the assumptions are sufficient for an answer. Indeed, it is better to have a question in mind first because that can guide both the choice of assumptions and the flavor of the model that is used. Sensing which assumptions might be critical and which irrelevant to the question at hand is the art of modeling and, for this, there is no substitute for a deep understanding of the biology. Good model building is a subjective exercise, dependent on local information and expertise, and contingent upon current knowledge. As to what biological insights all this might bring, that is best revealed by example.

## Three models

The examples that follow extend from cell biology to immunology to developmental biology. They are personal favorites and illuminate different issues.

### Learning how to think about non-identical compartments

The eukaryotic cell has an internal structure of membrane-bounded compartments—nucleus, endoplasmic reticulum, Golgi and endosomes—which dynamically interact through vesicle trafficking. Vesicles bud from and fuse to compartments, thereby exchanging lipids and proteins. The elucidation of trafficking mechanisms was celebrated in the 2013 Nobel Prize in Physiology or Medicine awarded to Jim Rothman, Randy Schekman and Thomas Sudhof. A puzzling question that remains unanswered is how distinct compartments remain distinct, with varied lipid and protein profiles, despite continuously exchanging material. How are non-identical compartments created and maintained?

**Figure 1:**
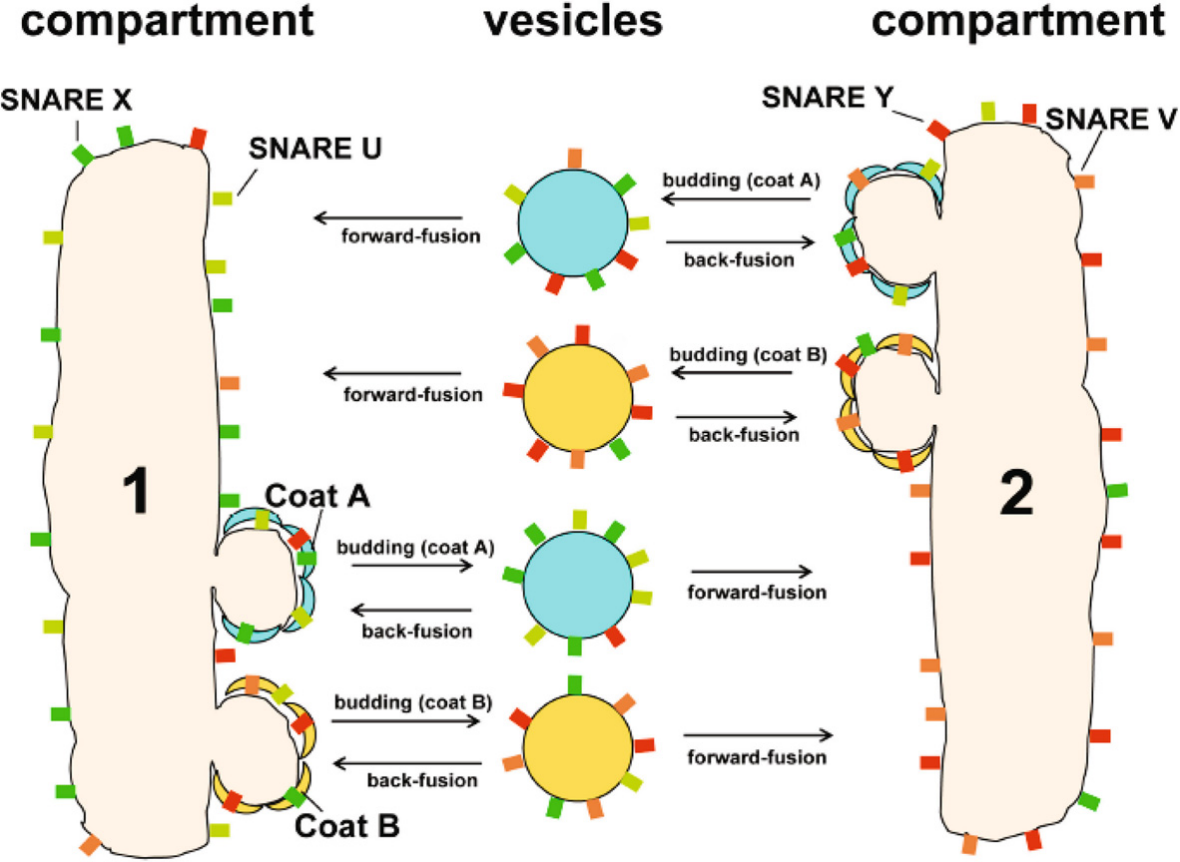
**Creation of non-identical compartments.** Schematic of the Heinrich–Rapoport model, from [23, Figure one], with the distribution of SNAREs corresponding approximately to the steady state with non-identical compartments. Ⓒ2005 Heinrich and Rapoport. Originally published in *Journal of Cell Biology*, **168:**271-280, doi:10.1083/jcb.200409087. SNARE, soluble N-ethyl-maleimide-sensitive factor attachment protein receptor.

Reinhart Heinrich and Tom Rapoport address this question through a mathematical model [23], which formalizes the sketch in Figure 1. Coat proteins A and B, corresponding to Coat Protein I (COPI) and COPII, encourage vesicle budding from compartments 1 and 2. Soluble N-ethyl-maleimide-sensitive factor attachment protein receptors (SNAREs) X, U, Y and V are present in the compartment membranes and mediate vesicle fusion by pairing X with U and Y with V, corresponding to v- and t-SNAREs. A critical assumption is that SNAREs are packaged into vesicles to an extent that depends on their affinities for coats, for which there is some experimental evidence. If the cognate SNAREs X and U bind better to coat A than to coat B, while SNAREs Y and V bind better to coat B than to coat A, then the model exhibits a threshold in the relative affinities at which non-identical compartments naturally emerge. Above this threshold, even if the model is started with identical distributions of SNAREs in the two compartments, it evolves over time to a steady state in which the SNARE distributions are different. This is illustrated in Figure 1, with a preponderance of SNAREs X and U in compartment 1 and a preponderance of SNAREs Y and V in compartment 2.

The actual details of coats and SNAREs are a good deal more complicated than in this model. It is a parsimonious model, containing just enough biological detail to reveal the phenomenon, thereby allowing its essence—the differential affinity of SNAREs for coats—to be clearly understood. We see that a model can be useful not just to account for data—there is no data here—but to help us think. However, the biological details are only part of the story; the mathematical details must also be addressed. Even a parsimonious model typically has several free parameters, such as, in this case, binding affinities or total amounts of SNAREs or coats. To sidestep the parameter problem, discussed further in the next example, parameters of a similar type are set equal to each other. Here, judgment plays a role in assessing that differences in these parameters might play a secondary role. The merit of this assumption could have been tested by sensitivity analysis [24], which can offer reassurance that the model behavior is not some lucky accident of the particular values chosen for the parameters.

The model immediately suggests experiments that could falsify it, of which the most compelling would be *in vitro* reconstitution of compartments with a minimal set of coats and SNAREs. I was curious about whether this had been attempted and asked Tom Rapoport about it. Tom is a cell biologist [25] whereas the late Reinhart Heinrich was a physicist [26]. Their long-standing collaboration (they were pioneers in the development of metabolic control analysis in the 1970s) was stimulated by Tom’s father, Samuel Rapoport, himself a biochemist with mathematical convictions [27]. Tom explained that the model had arisen from his sense that there might be a simple explanation for distinct compartments, despite the complexity of trafficking mechanisms, but that his own laboratory was not in a position to undertake the follow-up experiments. Although he had discussed the ideas with others who were better placed to do so, the field still seemed to be focused on the molecular details.

The model makes us think further, as all good models should. The morphology of a multicellular organism is a hereditary feature that is encoded in DNA, in genetic regulatory programs that operate during development. But what encodes the morphology of the eukaryotic cell itself? This is also inherited: internal membranes are dissolved or fragmented during cell division, only to reform in their characteristic patterns in the daughter cells after cytokinesis. Trafficking proteins are genetically encoded but how is the information to reform compartments passed from mother to daughter? The Heinrich–Rapoport model suggests that this characteristic morphology may emerge dynamically, merely as a result of the right proteins being present along with the right lipids. This would be a form of epigenetic inheritance [28], in contrast to the usual genetic encoding in DNA. Of course, DNA never functions on its own, only in concert with a cell. The Heinrich–Rapoport model reminds us that the cell is the basic unit of life. Somebody really ought to test the model.

### Discrimination by the T-cell receptor and the parameter problem

Cytotoxic T cells of the adaptive immune system discriminate between self and non-self through the interaction between the T-cell receptor (TCR) and major histocompatibility complex (MHC) proteins on the surface of a target cell. MHCs present short peptide antigens (eight amino acids), derived from proteins in the target cell, on their external surface. The discrimination mechanism must be highly sensitive, to detect a small number of strong agonist, non-self peptide-MHCs (pMHCs) against a much larger background of weak agonist, self pMHCs on the same target cell. It must also be highly specific, since the difference between strong- and weak-agonist pMHCs may rest on only a single amino acid. Discrimination also appears to be very fast, with downstream signaling proteins being activated within 15 seconds of TCR interaction with a strong agonist pMHC. A molecular device that discriminates with such speed, sensitivity and specificity would be a challenge to modern engineering. It is an impressive demonstration of evolutionary tinkering, which Grégoire Altan-Bonnet and Ron Germain sought to explain by combining mathematical modeling with experiments [29].

**Figure 2:**
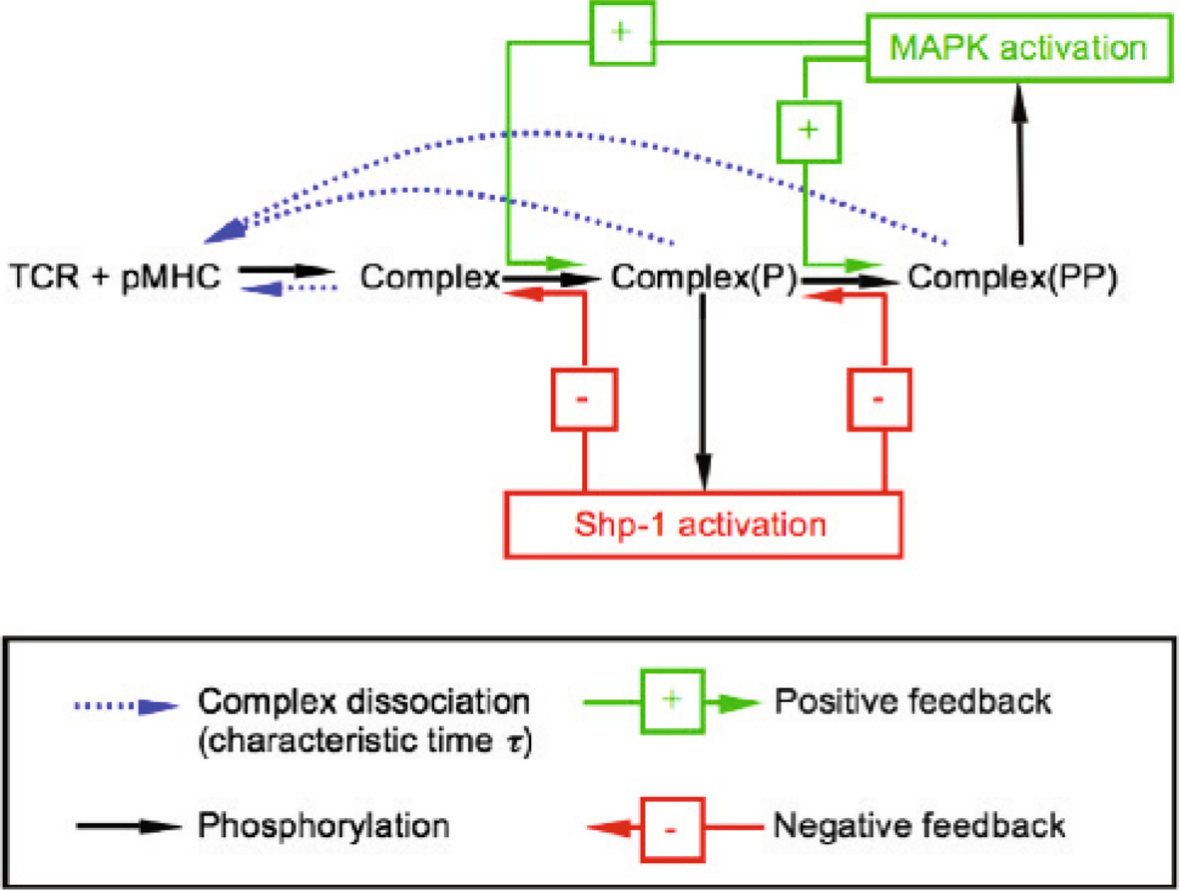
**Discrimination by the T-cell receptor.** Schematic of the Altan-Bonnet–Germain model from [29, Figure two A], showing a kinetic proofreading scheme through a sequence of tyrosine phosphorylations, which is triggered by the binding of the TCR to pMHC, linked with a negative feedback loop through the tyrosine phosphatase SHP-1 and a positive feedback loop through MAPK. MAPK, mitogen-activated protein kinase; pMHC, peptide-major histocompatibility complex; P, singly phosphorylated; PP, multiply phosphorylated; SHP-1, SH2 domain-containing tyrosine phosphatase-1; TCR, T-cell receptor.

The lifetime of pMHC-TCR binding had been found to be one of the few biophysical quantities to correlate with T-cell activation. Specificity through binding had previously been analyzed by John Hopfield in a classic study [30]. He showed that a system at thermodynamic equilibrium could not achieve discrimination beyond a certain minimum level but that with sufficient dissipation of energy, arbitrarily high levels of discrimination were possible. He suggested a ‘kinetic proofreading’ scheme to accomplish this, which Tim McKeithan subsequently extended to explain TCR specificity [31]. pMHC binding to the TCR activates lymphocyte-specific protein tyrosine kinase (LCK), which undertakes multiple phosphorylations of TCR accessory proteins and these phosphorylations are presumed to be the dissipative steps. However, the difficulty with a purely kinetic proofreading scheme is that specificity is purchased at the expense of both sensitivity and speed [32]. Previous work from the Germain laboratory had implicated SH2 domain-containing tyrosine phosphatase-1 (SHP-1) in downregulating LCK for weak agonists and the mitogen-activated protein kinase (MAPK), extracellular signal-regulated kinase (ERK), in inhibiting SHP-1 for strong agonists [33]. This led Altan-Bonnet and Germain to put forward the scheme in Figure 2, in which a core kinetic proofreading scheme stimulates negative feedback through SHP-1 together with a slower positive feedback through ERK. The behavior of interlinked feedback loops has been a recurring theme in the literature [34,35].

A parsimonious model of such a system might have been formulated with abstract negative and positive feedback differentially influencing a simple kinetic proofreading scheme. In fact, exactly this was done some years later [36]. The advantage of such parsimony is that it is easier to analyze how the interaction between negative and positive feedback regulates model behavior. The biological wood starts to emerge from the molecular trees, much as it did for Heinrich and Rapoport in the previous example. But the goal here also involves the interpretation of quantitative experimental data. Altan-Bonnet and Germain opted instead for a detailed model based on the known biochemistry. Their model has around 300 dynamical variables. Only the core module is described in the main paper, with the remaining nine modules consigned to the Supplementary Graveyard. Herbert Sauro’s JDesigner software, part of the Systems Biology Workbench [37], is required to view the model in its entirety.

The tension between parsimony and detail runs through systems biology like a fault line. To some, and particularly to experimentalists, detail is verisimilitude. The more a model looks like reality, the more it might tell us about reality. The devil is in the details. But we never bother ourselves with all the details. All those phosphorylation sites? Really? All 12 subunits of RNA Pol II? Really? We are always simplifying—ignoring what we think is irrelevant—or abstracting—replacing something complicated by some higher-level entity that is easier to grasp. This is as true for the experimentalist’s informal model—the cartoon that is sketched on the whiteboard—as it is for the mathematician’s formal model. It is impossible to think about molecular systems without such strategies: it is just that experimentalists and mathematicians do it differently and with different motivations. There is much to learn on both sides, for mathematicians about the hidden assumptions that guide experimental thinking, often so deeply buried as to require psychoanalysis to elicit, and for experimentalists about the power of abstraction and its ability to offer a new language in which to think. We are in the infancy of learning how to learn from each other.

The principal disadvantage of a biologically detailed model is the attendant parameter problem. Parameter values are usually estimated by fitting the model to experimental data. Fitting only constrains some parameters; a good rule of thumb is that 20% of the parameters are well constrained by fitting, while 80% are not [38]. As John von Neumann said, expressing a mathematician’s disdain for such sloppiness, ‘With four parameters I can fit an elephant and with five I can make him wiggle his trunk’ [39]. What von Neumann meant is that a model with too many parameters is hard to falsify. It can fit almost any data and what explanatory power it might have may only be an accident of the particular parameter values that emerge from the fitting procedure. Judging from some of the literature, we seem to forget that a model does not predict the data to which it is fitted: the model is chosen to fit them. In disciplines where fitting is a professional necessity, such as X-ray crystallography, it is standard practice to fit to a training data set and to falsify the model, once it is fitted, on whether or not it predicts what is important [40]. In other words, do not fit what you want to explain!

Remarkably, Altan-Bonnet and Germain sidestepped these problems by not fitting their model at all. They adopted the same tactic as Heinrich and Rapoport and set many similar parameters to the same value, leaving a relatively small number of free parameters. Biological detail was balanced by parametric parsimony. The free parameters were then heroically estimated in independent experiments. I am told that every model parameter was constrained, although this is not at all clear from the paper.

What was also not mentioned, as Ron Germain reported, is that ‘the model never worked until we actually measured ERK activation at the single cell level and discovered its digital nature’. We see that the published model emerged through a cycle of falsification, although here it is the model that falsifies the interpretation of population-averaged data, reminding us yet again that the mean may not be representative of the distribution.

With the measured parameter values, the model exhibits a sharp threshold at a pMHC-TCR lifetime of about 3 seconds, above which a few pMHCs (10 to 100) are sufficient to trigger full downstream activation of ERK in 3 minutes. Lifetimes below the threshold exhibit a hierarchy of responses, with those close to the threshold triggering activation only with much larger amounts of pMHCs (100,000), while those further below the threshold are squelched by the negative feedback without ERK activation. This accounts well for the specificity, sensitivity and speed of T-cell discrimination but the authors went further. They interrogated the fitted model to make predictions about issues such as antagonism and tunability and they confirmed these with new experiments [29]. The model was repeatedly forced to put its falsifiability on the line. In doing so, the boundary of its explanatory power was reached: it could not account for the delay in ERK activation with very weak ligands and the authors explicitly pointed this out. This should be the accepted practice; it is the equivalent of a negative control in an experiment. A model that explains everything, explains nothing. Even von Neumann might have approved.

To be so successful, a detailed model relies on a powerful experimental platform. The OT-1 T cells were obtained from a transgenic mouse line that only expresses a TCR that is sensitive to the strong-agonist peptide SIINFEKL (amino acids 257 to 264 of chicken ovalbumin). The RMA-S target cells were derived from a lymphoma that was mutagenized to be deficient in antigen processing, so that the cells present only exogenously supplied peptides on MHCs. T-cell activation was measured by flow cytometry with a phospho-specific antibody to activated ERK. In this way, calibrated amounts of chosen peptides can be presented on MHCs to a single type of TCR, much of the molecular and cellular heterogeneity can be controlled and quantitative data obtained at the single-cell level. Such exceptional experimental capabilities are not always available in other biological contexts.

### From micro to macro: the somitogenesis clock

**Figure 3:**
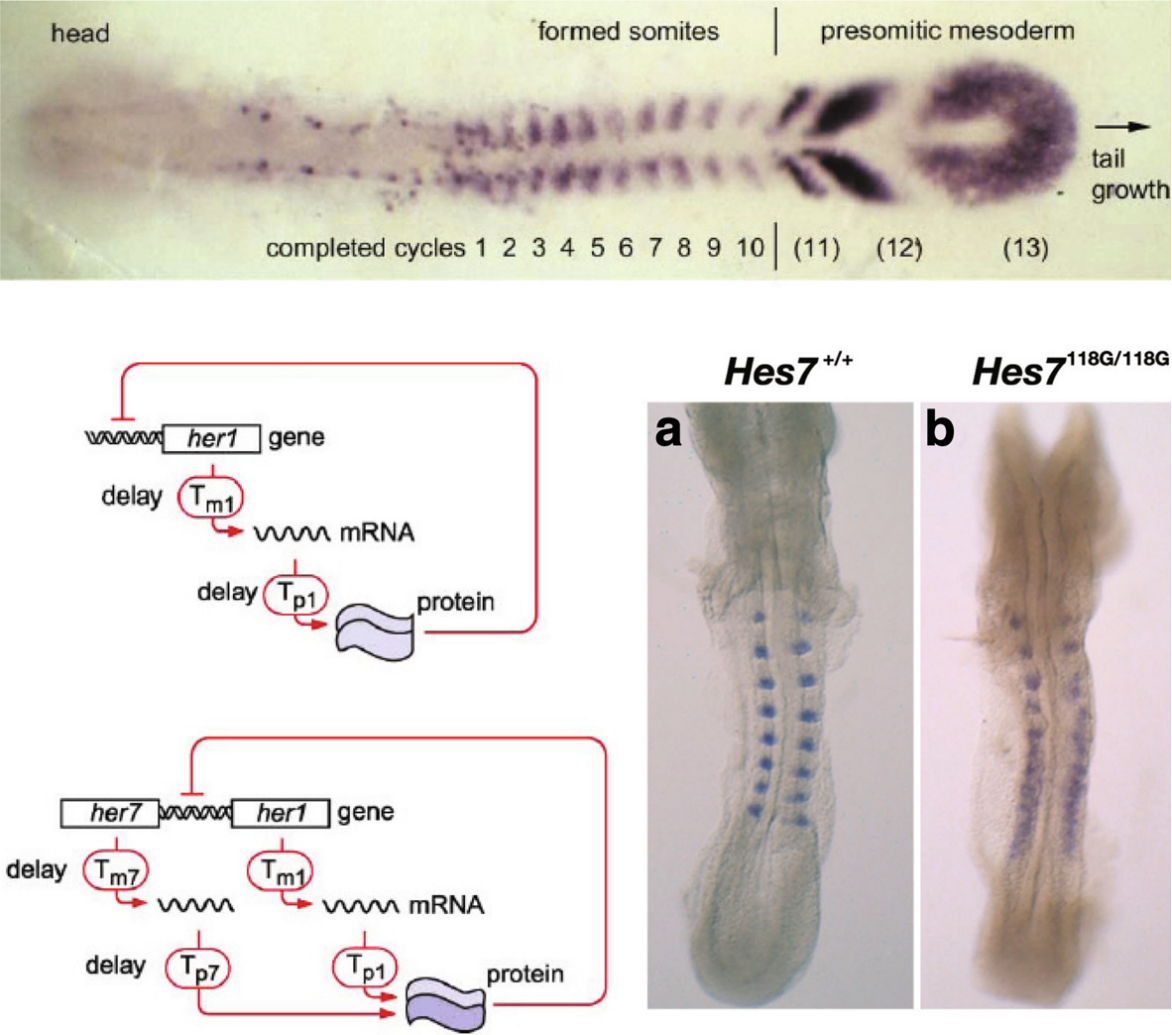
**The somitogenesis clock.** Top: A zebrafish embryo at the ten-somite stage, stained by *in situ* hybridization for mRNA of the Notch ligand DeltaC, taken from [47, Figure one]. Bottom left: Potential auto-regulatory mechanisms in the zebrafish, taken from [47, Figure three A,B]. In the upper mechanism, the Her1 protein dimerizes before repressing its own transcription. In the lower mechanism, Her1 and Her7 form a heterodimer, which represses transcription of both genes, which occur close to each other but are transcribed in opposite directions. Explicit transcription and translation delays are shown, which are incorporated in the corresponding models. Bottom right: Mouse embryos stained by *in situ* hybridization for Uncx4.1 mRNA, a homeobox gene that marks somites, taken from [52, Figure four].

Animals exhibit repetitive anatomical structures, like the spinal column and its attendant array of ribs and muscles in vertebrates and the multiple body segments carrying wings, halteres and legs in arthropods like *Drosophila*. During vertebrate development, repetitive structures form sequentially over time. In the mid 1970s, the developmental biologist Jonathan Cooke and the mathematician Chris Zeeman suggested that the successive formation of somites (bilateral blocks of mesodermal tissue on either side of the neural tube—see Figure 3) might be driven by a cell-autonomous clock, which progressively initiates somite formation in an anterior to posterior sequence as if in a wavefront [41]. They were led to this clock-and-wavefront model in an attempt to explain the remarkable consistency of somite number within a species, despite substantial variation in embryo sizes at the onset of somitogenesis [42]. In the absence of molecular details, which were beyond reach at the time, their idea fell on stony ground. It disappeared from the literature until Olivier Pourquié’s group found the clock in the chicken. His laboratory showed, using fluorescent *in situ* hybridization to mRNA in tissue, that the gene *c-hairy1* exhibits oscillatory mRNA expression with a period of 90 minutes, exactly the time required to form one somite [43]. The somitogenesis clock was found to be conserved across vertebrates, with basic helix-loop-helix transcription factors of the Hairy/Enhancer of Split (HES) family, acting downstream of Notch signaling, exhibiting oscillations in expression with periods ranging from 30 minutes in zebrafish (at 28°C) to 120 minutes in mouse [44]. Such oscillatory genes in somite formation were termed cyclic genes.

As to the mechanism of the oscillation, negative feedback of a protein on its own gene was known to be a feature of other oscillators [45] and some cyclic genes, like *hes7* in the mouse, were found to exhibit this property. Negative feedback is usually associated with homeostasis—with restoring a system after perturbation—but, as engineers know all too well, it can bring with it the seeds of instability and oscillation [46]. However, Palmeirim *et al.* had blocked protein synthesis in chick embryos with cycloheximide and found that *c-hairy1* mRNA continued to oscillate, suggesting that *c-hairy1* was not itself part of a negative-feedback oscillator but was, perhaps, driven by some other oscillatory mechanism. It remained unclear how the clock worked.

The developmental biologist Julian Lewis tried to resolve this question in the zebrafish with the help of a mathematical model [47]. Zebrafish have a very short somite-formation period of 30 minutes, suggesting that evolutionary tinkering may have led to a less elaborate oscillator than in other animals. The HES family genes *her1* and *her7* were known to exhibit oscillations and there was some evidence for negative auto-regulation.

Lewis opted for the most parsimonious of models to formalize negative auto-regulation of *her1* and *her7* on themselves, as informally depicted in Figure 3. However, he made one critical addition by explicitly incorporating the time delays in transcription and translation. Time delay in a negative feedback loop is one feature that promotes oscillation, the other being the strength of the negative feedback. Indeed, there seems to be a trade-off between these features: the more delay, the less strong the feedback has to be for oscillation to occur [48]. Lewis acknowledged the mathematical biologist Nick Monk for alerting him to the importance of delays and Lewis’s article in *Current Biology* appeared beside one from Monk exploring time delays in a variety of molecular oscillators [49]. The idea must have been in the air because Jensen *et al.* independently made the same suggestion in a letter [50].

The model parameters, including the time delays, were all estimated on the basis of reasonable choices for *her1* and *her7*, taking into account, for instance, the intronic structure of the genes to estimate transcriptional time delays. Nothing was fitted. With the estimated values, the models showed sustained periodic oscillations. A pure Her7 oscillator with homodimerization of Her7 prior to DNA binding (which determines the strength of the repression) had a period of 30 minutes. As with the Heinrich–Rapoport model, there is no data but much biology. What is achieved is the demonstration that a simple auto-regulatory loop can plausibly yield sustained oscillations of the right period. A significant finding was that the oscillations were remarkably robust to the rate of protein synthesis, which could be lowered by 90% without stopping the oscillations or, indeed, changing the period very much. This suggests a different interpretation of Palmeirim *et al.*’s cycloheximide block in the chick. As Lewis pointed out, ‘in studying these biological feedback phenomena, intuition without the support of a little mathematics can be a treacherous guide’, a theme to which he returned in a later review [51].

A particularly startling test of the delay model was carried out in the mouse by Ryoichiro Kageyama’s laboratory in collaboration with Lewis [52]. The period for somite formation in the mouse is 120 minutes and evidence had implicated the mouse *hes7* gene as part of the clock mechanism. Assuming a Hes7 half-life of 20 minutes (against a measured half-life of 22.3 minutes), Lewis’s delay model yielded sustained oscillations with a period just over 120 minutes. The model also showed that if Hes7 was stabilized slightly to have a half-life only 10 minutes longer, then the clock broke: the oscillations were no longer sustained but damped out after the first three or four peaks of expression [52, Figure six B]. Hirata *et al.* had the clever idea of mutating each of the seven lysine residues in Hes7 to arginine, on the basis that the ubiquitin-proteasomal degradation system would use one or more of these lysines for ubiquitination. The K14R mutant was found to repress *hes7* transcription to the same extent as the wild type but to have an increased half-life of 30 minutes. A knock-in mouse expressing Hes7 ^*K*14*R*/*K*14*R*^ showed, exactly as predicted, the first three to four somites clearly delineated, followed by a disorganized blur (Figure 3).

Further work from the Kageyama laboratory, as well as by others, has explored the role of introns in determining the transcriptional delays in the somitogenesis clock, leading to experiments in transgenic mice that again beautifully confirm the predictions of the Lewis model [53-55]. These results strongly suggest the critical role of delays in breaking the clock but it remains of interest to know the developmental consequences of a working clock with a different period to the wild type [56].

On the face of it, Julian Lewis’s simple model has been a predictive triumph. I cannot think of any other model that can so accurately predict what happens in re-engineered mice. On closer examination, however, there is something distinctly spooky about it. If mouse pre-somitic mesodermal cells are dissociated in culture, individual cells show repetitive peaks of expression of cyclic genes but with great variability in amplitude and period [57]. In isolation, the clock is noisy and unsynchronized, nothing like the beautiful regularity that is observed in the intact tissue. The simple Lewis model can be made much more detailed to allow for such things as stochasticity in gene expression, additional feedback and cell-to-cell communication by signaling pathways, which can serve to synchronize and entrain individual oscillators [47,58-60]. A more abstract approach can also be taken, in which emergent regularity is seen to arise when noisy oscillators interact through time-delayed couplings [61,62]. As Andy Oates said to me, such an abstraction ‘becomes simpler (or at least more satisfying) than an increasingly large genetic regulatory network, which starts to grow trunks at alarming angles’. These kinds of ‘tiered models’ have yielded much insight into the complex mechanisms at work in the tissue [63]. The thing is, none of this molecular complexity is present in the Lewis model. Yet, it describes what happens in the mouse with remarkable accuracy. The microscopic complexity seems to have conspired to produce something beautifully simple at the macroscopic level. In physics, the macroscopic gas law, *PV*=*RT*, is beautifully simple and statistical mechanics shows how it emerges from the chaos of molecular interactions [64]. How does the Lewis model emerge in the tissue from the molecular complexity within? It is as if we are seeing a tantalizing glimpse of some future science whose concepts and methods remain barely visible to us in the present. Every time I think about it, the hairs on the back of my neck stand up.

## Conclusion

A mathematical model is a logical machine for converting assumptions into conclusions. If the model is correct and we believe its assumptions then we must, as a matter of logic, believe its conclusions. This logical guarantee allows a modeler, in principle, to navigate with confidence far from the assumptions, perhaps much further than intuition might allow, no matter how insightful, and reach surprising conclusions. But, and this is the essential point, the certainty is always relative to the assumptions. Do we believe our assumptions? We believe fundamental physics on which biology rests. We can deduce many things from physics but not, alas, the existence of physicists. This leaves us, at least in the molecular realm, in the hands of phenomenology and informed guesswork. There is nothing wrong with that but we should not fool ourselves that our models are objective and predictive, in the sense of fundamental physics. They are, in James Black’s resonant phrase, ‘accurate descriptions of our pathetic thinking’.

Mathematical models are a tool, which some biologists have used to great effect. My distinguished Harvard colleague, Edward Wilson, has tried to reassure the mathematically phobic that they can still do good science without mathematics [65]. Absolutely, but why not use it when you can? Biology is complicated enough that we surely need every tool at our disposal. For those so minded, the perspective developed here suggests the following guidelines: 

1. *Ask a question.* Building models for the sake of doing so might keep mathematicians happy but it is a poor way to do biology. Asking a question guides the choice of assumptions and the flavor of model and provides a criterion by which success can be judged.

2. *Keep it simple.* Including all the biochemical details may reassure biologists but it is a poor way to model. Keep the complexity of the assumptions in line with the experimental context and try to find the right abstractions.

3. *If the model cannot be falsified, it is not telling you anything.* Fitting is the bane of modeling. It deludes us into believing that we have predicted what we have fitted when all we have done is to select the model so that it fits. So, do not fit what you want to explain; stick the model’s neck out after it is fitted and try to falsify it.

In later life, Charles Darwin looked back on his early repugnance for mathematics, the fault of a teacher who was ‘a very dull man’, and said, ‘I have deeply regretted that I did not proceed far enough at least to understand something of the great leading principles of mathematics; for men thus endowed seem to have an extra sense’ [66]. One of those people with an extra sense was an Augustinian friar, toiling in the provincial obscurity of Austro-Hungarian Brünn, teaching physics in the local school while laying the foundations for rescuing Darwin’s theory from oblivion [67], a task later accomplished, in the hands of J. B. S. Haldane, R. A. Fisher and Sewall Wright, largely by mathematics. Darwin and Mendel represent the qualitative and quantitative traditions in biology. It is a historical tragedy that they never came together in their lifetimes. If we are going to make sense of systems biology, we shall have to do a lot better.

## Abbreviations

COP: Coat Protein I; ERK: Extracellular signal-regulated kinase; HES: Hairy/Enhancer of Split family; LCK: lymphocyte-specific protein tyrosine kinase; MAPK: mitogen-activated protein kinase; MHC: major histocompatibility complex; pMHC: peptide-MHC; SHP-1: SH2 domain-containing tyrosine phosphatase-1; SNARE: soluble N-ethyl-maleimide-sensitive factor attachment protein receptor; TCR: T-cell receptor.

## Competing interests

The author declares that he has no competing interests.
